# Anti-Inflammatory Potential of Extracellular Polysaccharide from the Moss Endophyte *Ovatospora brasiliensis* During Pathogen Infection

**DOI:** 10.3390/microorganisms13092037

**Published:** 2025-08-31

**Authors:** Jiayue Yang, Ying Sun, Mingchun Li, Qilin Yu

**Affiliations:** 1 National Key Laboratory of Intelligent Tracking and Forecasting for Infectious Diseases, College of Life Sciences, Nankai University, Tianjin 300071, China; tjjyyang@hotmail.com (J.Y.); 2120231588@mail.nankai.edu.cn (Y.S.); nklimingchun@163.com (M.L.); 2 Key Laboratory of Molecular Microbiology and Technology, Ministry of Education, Department of Microbiology, College of Life Sciences, Nankai University, Tianjin 300071, China

**Keywords:** systemic infection, anti-inflammation, fungal exopolysaccharide, moss endophyte, *Candida albicans*

## Abstract

Acute inflammation is frequently triggered by pathogen infections and contributes to host mortality. In this study, a new exopolysaccharide (ObEPS) was isolated from the moss endophyte *Ovatospora brasiliensis* and characterized for its structure and biological activity. Monosaccharide composition analysis revealed that ObEPS was mainly composed of galactose, glucose, mannose, and glucuronic acid. Multi-angle light scattering and conformation analysis showed a molar mass of 10^5^–10^6^ Da and a compact chain conformation. In vitro experiments showed that ObEPS markedly inhibited nitric oxide production and reduced pro-inflammatory cytokine expression in lipopolysaccharide (LPS)-stimulated RAW264.7 macrophages. In a systemic *Candida albicans* infection model, ObEPS combined with fluconazole significantly reduced fungal colony-forming units (CFUs)/g kidney from 3.8 × 10^5^ to 0.1 × 10^5^, with the reduction of pro-inflammatory cytokine levels and tissue damage compared with the EPS-free groups suffering from *C. albicans* infection. Overall, these findings indicate that ObEPS has potent anti-inflammatory activity and may serve as a promising natural adjunct for mitigating infection-associated inflammatory damage.

## 1. Introduction

Severe sepsis and septic shock are major causes of mortality and morbidity worldwide [1,2]. Studies have shown that, in addition to bacterial infections, fungal infections have also significantly contributed to the rising incidence of sepsis [3,4]. The global burden of fungal infections is becoming increasingly severe, with approximately 1.5 million deaths annually attributed to invasive fungal infections. Among these, the most common is *C. albicans* [5,6], causing invasive candidiasis in hospital settings and associated with high morbidity and mortality rates [7,8,9]. The World Health Organization (WHO) has classified *C. albicans* as a critical priority group in the fungal pathogens list [8,10,11]. Invasive candidiasis or candidemia caused by *C. albicans* typically occurs in immunocompromised or critically ill patients, spreading through the bloodstream and leading to systemic infections. These infections often result in increased *Candida* colonization in the kidneys and other organs [12,13,14]. Therefore, it exacerbates inflammatory responses.

Traditional antifungal therapies, typically involving azole drugs, can partly control *C. albicans* infections and inhibit its proliferation in organs. However, during the later stages of infection, the inflammatory storm triggers an acute systemic inflammatory response. The overactivation of the immune system enhances the activity of effector cells, such as macrophages and neutrophils, leading to the release of large amounts of pro-inflammatory factors (e.g., TNF-α, IL-1β, IL-6). This cascade causes tissue damage, multiple organ dysfunction syndrome (MODS), and septic shock [15], resulting in extremely high mortality rates. Therefore, an effective strategy to mitigate systemic fungal infections should include both controlling fungal proliferation and suppressing excessive inflammatory responses in the host [16,17]. Consequently, current antifungal treatments have limited effectiveness in suppressing the inflammatory storm and struggle to balance the dual needs of infection control and immune regulation [18]. There is an urgent need to develop novel, precise therapies to improve patient outcomes.

Natural polysaccharides are high-molecular-weight compounds primarily extracted from the cell walls and membranes of plants, fungi, algae, and bacteria [19,20]. An increasing number of studies have confirmed that polysaccharides exhibit excellent biocompatibility, biodegradability, low toxicity, and diverse biological activities, such as antioxidant, anti-inflammatory, immunomodulatory, and antimicrobial properties [20,21]. Fungal extracellular polysaccharides are considered one of the most important bioactive components in fungi [22,23,24]. Increasing studies have shown that these unique extracellular polysaccharides secreted by fungi often possess distinctive biological activities, including antitumor, antioxidant, immunomodulatory, anti-inflammatory, hepatoprotective, cardioprotective, and anti-aging properties [10,25]. For instance, crude extracellular polysaccharide from the endophytic fungus *P. citreonigrum* CSL-27 in saffron, can significantly attenuate gentamicin-induced HEI-OC1 cell damage and increase the survival rate of zebrafish cells [26]. Also, Zeng et al. reported the isolation of two polysaccharides, DGS1 and DGS2 (corresponding to CSL-27 DY2 and DG2), from *Fusarium solani* DO7 by solid-state fermentation. DGS1 and DGS2 differed in monosaccharide composition and glycosidic linkages. For example, DGS2 contains relatively higher proportions of (1→5)-Araf, (1→3)-Manp and (1→6)-Galp residues, which are chemical features associated with immunomodulatory effects. In RAW 264.7 macrophages, both polysaccharides significantly enhanced TNF-α, IL-6, and NO production without cytotoxicity, with DGS2 exhibiting the stronger immunostimulatory and antioxidant activity, suggesting a potential protective role in inflammation modulation [25]. In short, fungal extracellular polysaccharides could be used as potent immunomodulatory agents. Nevertheless, few polysaccharide-based therapies have been applied for the treatment of *C. albicans*, underscoring the critical need to explore polysaccharides with immunomodulatory and anti-inflammatory properties as potential therapeutic agents.

This study focuses on the immunomodulatory properties of the extracellular polysaccharides produced by the moss endophytic fungi *Ovatospora brasiliensis*. We aimed to characterize the monosaccharide composition, molecular weight, and functional groups of the *O. brasiliensis* polysaccharides (ObEPS). Furthermore, we evaluated their anti-inflammatory potential by assessing the regulation of inflammatory cytokines and nitric oxide (NO) production in lipopolysaccharide (LPS)-induced RAW264.7 macrophages in vitro. The immunomodulatory capacity of ObEPS was further assessed using a *C. albicans* systemic infection model in mice. During the systemic infection, ObEPS plus fluconazole (FCZ) treatment efficiently prolonged survival of mice and attenuated the inflammatory response induced by the *C. albicans* infection. This research first explores the anti-inflammatory potential of ObEPS and provides a new therapeutic strategy to mitigate the inflammatory storm caused by systemic fungal infections.

This study focused on the immunomodulatory properties of ObEPS produced by the moss endophytic fungus *Ovatospora brasiliensis*. We hypothesized that ObEPS, due to its unique chemical characteristics, can modulate inflammatory responses and may serve as a natural candidate for mitigating infection-associated inflammation. To test this hypothesis, we conducted a detailed structural characterization of ObEPS. Furthermore, we investigated its immunomodulatory potential in LPS-stimulated RAW264.7 macrophages in vitro, as well as in a systemic *C. albicans* infection model in vivo. This work aims to provide preliminary evidence supporting the potential role of ObEPS in regulating inflammatory responses and a new therapeutic strategy to mitigate the inflammatory storm in pathogen infections.

## 2. Materials and Methods

### 2.1. Materials and Reagents

All three bryophytes—*Philonotis lancifolia*, *Plagiomnium acutum*, and *Pogonatum inflexum*—were collected in Hunan, China. *O. brasiliensis* NK6015, *Trichoderma atroviride* NK1607, and *Penicillium javanicum* NK6001, which were used in the present work, were isolated from the bryophyte herbs and stored in Nankai University (Tianjin, China). Alcohol, phenol, chloroform, and normal butanol were purchased from Sinopharm Chemical Reagent Co. (Shanghai, China). All of the enzyme-linked immunosorbent assay kits (ELISA) were purchased from Shanghai Jonln Bioscience (Shanghai, China). The mice were purchased from Beijing HFK Bioscience (Beijing, China). FITC anti-mouse F4/80 (123107), FITC anti-mouse Ly-6G (127605), and APC anti-mouse/human CD11b (101212) were purchased from BioLegend (Biotechnology Co., Ltd., San Diego, CA, USA).

### 2.2. Isolation and Identification of Fungi from Moss Sample

Moss samples were collected and gently washed with sterile distilled water to remove surface debris. Small fragments were aseptically excised and surface-sterilized by immersion in 70% ethanol for 30 s, followed by 2% sodium hypochlorite solution for 1–2 min, and then rinsed three times with sterile water. The sterilized fragments were placed on potato dextrose agar (PDA) plates supplemented with antibiotics to inhibit bacterial growth. Plates were incubated at 28 °C for 5–7 days, and emerging fungal colonies were further inoculated onto fresh PDA plates for purification and subsequent identification.

### 2.3. Extraction and Purification of the Exopolysaccharides ObEPS

The extraction of exopolysaccharides (ObEPS) was performed according to the method described by Wang et al. [27]. After 7 days of fermentation, the culture broth was harvested and centrifuged at 12,000 rpm to remove cellular debris. The resulting supernatant was then subjected to protein removal using an equal volume (1:1, *v*/*v*) of Sevag reagent (chloroform: n-butanol = 4:1, *v*/*v*), with continuous stirring for 30 min. After standing for 60 min, the upper aqueous phase was carefully collected, and the Sevag treatment was repeated multiple times until no visible protein interface remained. Subsequently, the ObEPS-containing solution was transferred into a dialysis bag (molecular weight cut-off: 8–14 kDa; Beijing Dingguo Changsheng Biotechnology Co., Ltd., Beijing, China) and dialyzed against deionized water for 72 h, with the water being replaced every 8 h to ensure the thorough removal of salts and other small-molecule impurities. Finally, the deproteinized and dialyzed ObEPS solution was dried by a freezing vacuum lyophilizer (SCIENTZ-10N/A, Ningbo Xinzhi Biological Technology Co., Ltd., Ningbo, China) to obtain ObEPS powder without residual solvents.

### 2.4. Monosaccharide Composition Analysis

Approximately 5 mg of ObEPS was hydrolyzed with 2 M TFA at 121 °C for 2 h in a sealed tube. The hydrolysate was dried under a stream of nitrogen gas, washed with methanol, and dried again. This methanol washing step was repeated 2–3 times to remove residual acid. The residue was then dissolved in ultrapure water and filtered through a 0.22 μm microporous membrane before analysis. The monosaccharide composition was analyzed using a high-performance anion-exchange chromatography system equipped with a pulsed amperometric detector (HPAEC-PAD; ICS 5000+, Thermo Fisher Scientific, Waltham, MA, USA). Separation was achieved on a Dionex™ CarboPac™ PA-20 column (3 × 150 mm, 10 μm) at 30 °C. The injection volume was 5 μL and the flow rate was 0.5 mL/min. The elution gradient was programmed as follows: 0 min, A/B/C (95:5:0); 26 min, A/B/C (85:5:10); 42 min, A/B/C (85:5:10); 42.1 min, A/B/C (60:0:40); 52 min, A/B/C (60:40:0); 52.1 min, A/B/C (95:5:0); 60 min, A/B/C (95:5:0). Chromatographic data were processed using Chromeleon 7.2 software (Thermo Fisher Scientific, Waltham, MA, USA). Monosaccharides were identified based on the retention times of corresponding standards, and their concentrations were quantified using external standard calibration curves. The following monosaccharides, including fucose, rhamnose, arabinose, galactose, glucose, xylose, mannose, fructose, ribose, galacturonic acid, glucuronic acid, mannuronic acid, and guluronic acid, were used as standards in the HPLC analysis.

### 2.5. Molecular Weight and Conformation Analysis of ObEPS by SEC-MALS

#### 2.5.1. Sample Preparation

ObEPS was dissolved in 0.1 M NaNO_3_ aqueous solution containing 0.02% NaN_3_ at a final concentration of 1 mg/mL. The solution was filtered through a 0.45 μm pore-size membrane filter prior to analysis to remove insoluble particles and ensure sample homogeneity.

#### 2.5.2. Instrumentation

The molecular weight and conformation of ObEPS were determined using a size-exclusion chromatography system (SEC) coupled with a multi-angle laser light scattering detector (MALS) and a differential refractive index detector (RI). The system consisted of the following: HPLC system: UltiMate 3000 (Thermo Fisher Scientific, USA); MALS detector: DAWN HELEOS-II (Wyatt Technology, Goleta, CA, USA); RI detector: Optilab T-rEX (Wyatt Technology, Goleta, CA, USA); columns: two SEC columns (Shodex OHpak SB-805 HQ and OHpak SB-803 HQ, 300 × 8 mm, Showa Denko, Tokyo, Japan), connected in series. The column temperature was maintained at 45 °C, the injection volume was 100 μL, and the mobile phase was 0.1 M NaNO_3_ containing 0.02% NaN_3_, with an isocratic flow rate of 0.6 mL/min for 75 min.

#### 2.5.3. Calibration and dn/dc Determination

The MALS detector was normalized using toluene, and detector alignment was verified using bovine serum albumin (BSA, 66 kDa) as a standard. The differential refractive index increment (dn/dc) of ObEPS in 0.1 M NaNO_3_ containing 0.02% NaN_3_ was determined in-line using the Optilab T-rEX RI detector (Wyatt, IN, USA) and was measured to be 0.141 mL/g, which is consistent with typical values for polysaccharides.

#### 2.5.4. Data Analysis

Data were processed using ASTRA 6.1 software (Wyatt Technology). The number-average molecular weight (Mn), weight-average molecular weight (Mw), z-average molecular weight (Mz), polydispersity index (Mw/Mn), and root mean square (RMS) radius were calculated. Conformation plots were generated based on the relationship between molar mass and RMS radius to assess the molecular chain structure.

### 2.6. Nuclear Magnetic Resonance (NMR) Analysis

The ^1^H NMR spectra of the polysaccharide samples were recorded on a Bruker 600 MHz spectrometer (Bruker, Ettlingen, Germany). Samples were dissolved in deuterium oxide (D_2_O) at a concentration of approximately 10–20 mg/mL and transferred into 5 mm NMR tubes. To remove exchangeable protons, each sample was lyophilized from D_2_O three times prior to final dissolution. Spectra were acquired at 30 °C under standard acquisition parameters (spectral width 8012 Hz, relaxation delay 1.0 s, acquisition time 2.0 s). A total of 32 scans were collected for each spectrum to ensure adequate signal-to-noise ratios. Chemical shifts were referenced to the residual solvent peak of D_2_O at δ = 4.79 ppm for internal calibration. Data were processed using MestReNova software (version 14.3.0), applying zero-filling and exponential line broadening of 0.3 Hz prior to Fourier transformation.

### 2.7. Cell Culture

The RAW264.7 macrophages were cultured in high-glucose Dulbecco’s Modified Eagle’s Medium (DMEM; HyClone, Logan, UT, USA) supplemented with 10% heat-inactivated fetal bovine serum (FBS; HyClone, Logan, UT, USA) and maintained at 37 °C in a humidified atmosphere containing 5% CO_2_. RAW264.7 cells (5 × 10^6^ cells) were seeded into cell culture dishes containing the aforementioned medium. When the cells reached 80% confluence, they were sub-cultured continuously for subsequent experiments.

### 2.8. Macrophage Viability Assay

Macrophage viability was evaluated using the Cell Counting Kit-8 (CCK-8, Tianjin Simubiotech, Tianjin, China) according to the manufacturer’s instructions. Briefly, RAW264.7 cells were seeded into 96-well plates at a density of 5 × 10^3^ cells per well and allowed to adhere for 12 h. After adherence, the cells were treated with various concentrations of ObEPS (0, 25, 50, 100, and 200 μg/mL) for an additional 12 h. Following treatment, 10 μL of CCK-8 reagent was added to each well, and the plates were incubated at 37 °C in a 5% CO_2_ atmosphere for 1 h. The absorbance at 450 nm was then measured using a microplate reader (Enspire Multimode Plate reader, PerkinElmer LLC, Shelton, CT, USA).

### 2.9. NO Assay

RAW264.7 cells were seeded in 12-well plates at a density of 2 × 10^5^ cells per well and cultured for 12 h. After adherence, the cells were treated with 1 μg/mL LPS (added at the same final concentration across all groups) together with different concentrations of ObEPS (0, 25, 50, 100, and 200 μg/mL) for 12 h [28]. For the LPS control group, only LPS (1 μg/mL) was added, while for the LPS + EPS groups, the same concentration of LPS (1 μg/mL) was supplemented with the indicated concentrations of ObEPS. After treatment, the culture supernatant was collected and aliquoted into a 96-well plate at 50 μL per well. Subsequently, 50 μL of Griess Reagent I (pre-equilibrated to room temperature) was added to each well, followed by the addition of 50 μL of Griess Reagent II (also at room temperature). The absorbance was measured at 540 nm using a microplate reader. A group with no LPS or ObEPS added was used as a control in the following tests.

### 2.10. Animals

This study was conducted in strict accordance with the guidelines approved by the Animal Care and Use Committee of Nankai University (Approve number 2024-SYDWLL-000297). Pathogen-free, 5-week-old female ICR mice weighing between 18 g and 20 g, were chosen and randomly divided into four groups with 10 replicates. All mice were maintained in an environment of constant temperature and regular 12 h day/12 h night cycles, with free access to food and water. All animal experiments were performed under protocols approved by the Animal Care and Use Committee of Nankai University.

### 2.11. C. albicans Systemic Infection Model

To assess the anti-inflammatory effects of ObEPS, the mice (excluding those in the control group) were intravenously injected with fresh *C. albicans* SC5314 cells (5 × 10^6^ cells/mouse) via the tail vein on day 1 to induce infection. Treatment began on day 2, during which the infected mice received either fluconazole alone or a combination of ObEPS and FCZ (ObEPS + FCZ), administered intravenously based on their assigned groups. Survival was tracked daily from day 0 through day 14. On day 9, three mice from each group were sacrificed; their kidneys were aseptically removed and weighed. The tissues were then homogenized in sterile PBS, and the fungal load was determined by colony-forming unit (CFU) assays using yeast extract peptone dextrose medium (YPD) plates.

### 2.12. RNA Extraction and Quantitative Real-Time Polymerase Chain Reaction (RT-qPCR)

RNA was extracted from kidney tissues or RAW264.7 cells with TRIzol reagent (Transgen Biotechnology, Beijing, China). Subsequently, cDNA was synthesized from RNA using a reverse transcription kit (Vazyme, Shanghai, China), and RT-qPCR was performed using SYBR Green Master Mix (Transegen Biotechnology, Beijing, China) according to the manufacturer’s instructions. The primer sequences (TsingKe Biological Technology, Beijing, China) are listed in Table S1. The program for RT-qPCR is listed in Table S2. Relative mRNA expression levels of IL-1β, TNF-α, IL-6, IFN-γ, NOX2, Arg1, iNOS were normalized to that of the housekeeping gene β-actin and calculated by the 2^−∆∆Ct^ method.

### 2.13. Enzyme-Linked Immunosorbent Assay (ELISA)

On day 8, the mice were sacrificed, and blood was collected from the eyeballs into anticoagulant tubes. The mice were then euthanized. The collected blood was centrifuged at 10,000 rpm for 5 min at 4 °C, and the supernatant serum was collected for further experiments. The levels of IL-1β and IFN-γ in the serum were measured using a mouse ELISA kit according to the manufacturer’s instructions.

### 2.14. Flow Cytometry Assay

On day 8 post-systemic infection, the kidneys of the mice were harvested. The renal tissues were processed and digested into single-cell suspensions, followed by two washes with phosphate-buffered saline (PBS) and resuspension. The cells were diluted at a ratio of 1:1000, stained with fluorescent dyes, and incubated in the dark at 4 °C for 30 min. After two additional washes with PBS, the cells were resuspended and subjected to flow cytometry analysis.

### 2.15. PAS Staining

PAS staining was performed using the Periodic Acid–Schiff Staining Kit (Beyotine, Beijing, China) according to the manufacturer’s instructions. After oxidation with periodic acid, the samples were stained with Schiff reagent and counterstained with hematoxylin, followed by mounting. The stained kidney sections were then observed and photographed under a microscope.

### 2.16. Hematoxylin and Eosin (H&E) Staining

Tissue sections were deparaffinized in xylene and rehydrated through a graded series of ethanol solutions. The slides were then stained with hematoxylin for 5 min, rinsed in running tap water, and differentiated in 1% acid alcohol. After bluing in running water, sections were counterstained with eosin for 1–3 min, dehydrated through ascending grades of ethanol, cleared in xylene, and mounted with a coverslip.

## 3. Results

### 3.1. Isolation and Characterization of Moss Endophytic Fungi Producing Anti-Inflammatory EPS

**Figure 1:**
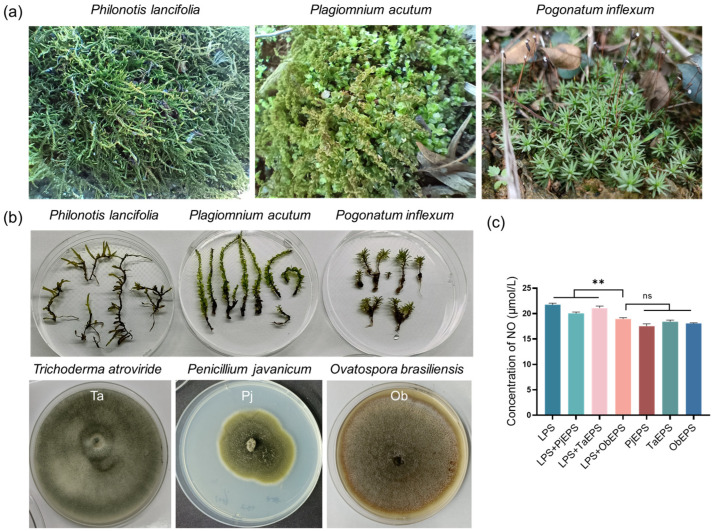
**Images of the mosses and corresponding endophytes producing EPS.** (**a**) Images of the wild mosses. (**b**) Images of the sampled mosses and corresponding endophytes. (**c**) Inhibition of NO production in LPS-treated RAW264.7 cells by the EPS. The asterisks (**) indicate significant difference in NO concentrations between the LPS + ObEPS group and other groups (*p* < 0.01), while the letters “ns” indicate no significant difference (*p* < 0.05).

To identify potential anti-inflammatory agents, we collected three species of wild mosses—*Philonotis lancifolia*, *Plagiomnium acutum*, and *Pogonatum inflexum*—from which endophytic fungi were isolated (Figure 1a,b). After cultivation and morphological identification, three fungal strains were obtained: *Trichoderma atroviride* NK1607 (Ta), *Penicillium javanicum* NK6001 (Pj), and *O. brasiliensis* NK6015 (Ob).

Exopolysaccharides (EPS) were extracted from the culture supernatants of these fungi and named TaEPS, PjEPS, and ObEPS, respectively. Their anti-inflammatory activity was preliminarily evaluated using an LPS-stimulated RAW264.7 cell model by measuring nitric oxide (NO) production. As shown in Figure 1c, the addition of ObEPS in the LPS-treated macrophages markedly reduced NO levels, whereas the addition of TaEPS or PjEPS had no significant impact on LPS-induced NO production. Meanwhile, in the absence of LPS, the macrophages treated by the three kinds of EPS exhibited similarly low NO levels. These results suggest that ObEPS had potential anti-inflammatory activity in the LPS-stressed condition.

### 3.2. Characterization of O. brasiliensis EPS

**Figure 2:**
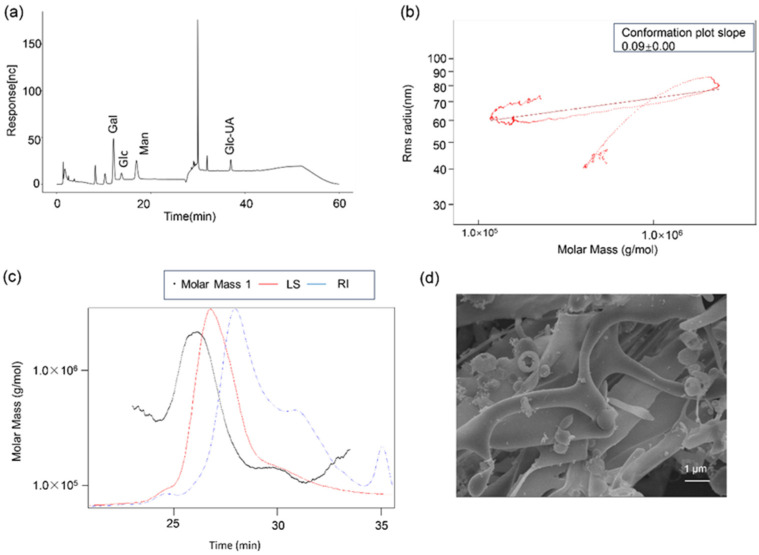
**Characterization of ObEPS.** (**a**) Monosaccharide composition. (**b**) Conformation plot slope. (**c**) Molar mass. (**d**) SEM image.

The characteristics of the exopolysaccharide (ObEPS) produced by *O. brasiliensis* were systematically analyzed. As shown in Figure 2a, the monosaccharide composition analysis indicated that ObEPS consisted primarily of galactose (Gal), glucose (Glc), mannose (Man), and glucuronic acid (GlcUA), suggesting a heteropolysaccharide nature. The analysis also revealed that the sample contained GalN (38.187 μg/mg) and GlcN (32.2247 μg/mg) (Table S3). Consistently, ^1^H-NMR analysis showed resonance at ~2.0 ppm, which could be attributed to the N-acetyl groups of the amino sugars (Figure S1). The conformation plot (Figure 2b) revealed a slope of 0.09, indicating a compact, near-spherical molecular conformation in solution. The molecular weight distribution, determined by SEC-MALS (Figure 2c), showed that ObEPS possessed a weight-average molar mass (Mw) at the order of 10^5^–10^6^ g/mol, with a relatively narrow distribution and a single main peak. The SEM image (Figure 2d) demonstrated that the dried ObEPS formed a dense, aggregated network structure with a rough and curled surface morphology, consistent with typical exopolysaccharide physical features. These structural features collectively imply that ObEPS has significant potential as a functional biopolymer. The presence of glucuronic acid and its compact conformation suggest favorable interactions with cellular membranes and the ability to modulate the local microenvironment, which may contribute to anti-inflammatory effects. Moreover, the dense network morphology and suitable molecular weight range make ObEPS an attractive candidate for the development of biocompatible material applications.

As shown in the ^1^H NMR spectrum of ObEPS (Figure S1), the dominant resonances are located in the 3.0–5.5 ppm region, which correspond to the ring protons (H1–H6) of the sugar residues. No broad or intense signals are observed in the aromatic region (6.5–8.5 ppm), where aromatic amino acid residues of proteins typically resonate. The small sharp peak at 7.28 ppm is of very low intensity and is more likely attributable to trace contaminants rather than protein-derived aromatic protons. In the aliphatic region (0.8–1.6 ppm), the minor peaks are consistent with possible methyl or acetyl substituents of the polysaccharide or trace low-molecular-weight impurities. No isolated signal in the 2.8–3.1 ppm region (often indicating lysine and arginine side chains) was detected. Moreover, the spectra are broad, and very few anomeric signals are observed compared to the ring proton signals. This suggests the possibility of amino sugars or O-acetylation in the sample. Nevertheless, we could not thoroughly exclude the presence of trace proteins in the ObEPS samples that also led to chemical shifts in the NMR spectra. However, the Bradford test showed that there is no detectable protein signal, excluding the possibility of high-level protein contamination in the ObEPS samples.

### 3.3. Anti-Inflammatory Effect and Cytocompatibility of ObEPS in Macrophages

**Figure 3:**
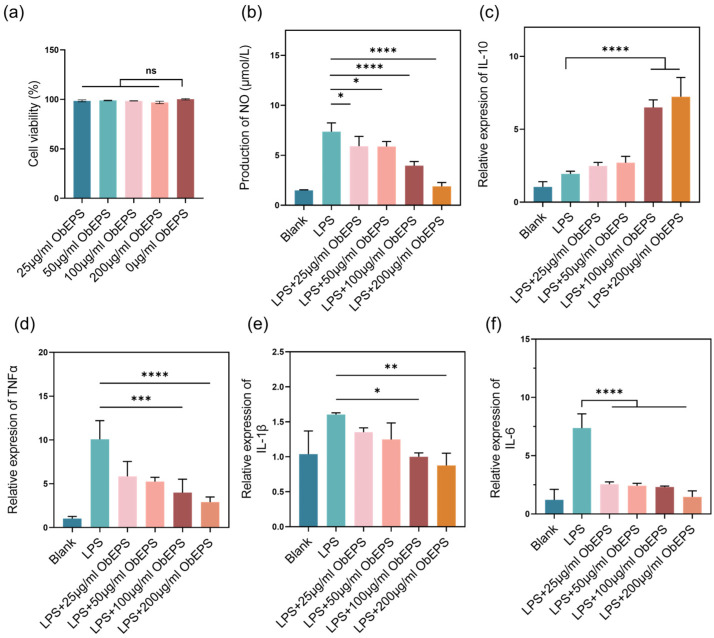
**Anti-inflammatory effect of ObEPS in the RAW264.7 macrophages.** (**a**) Cell viability of the macrophages after 6 h of ObEPS treatment at different concentrations. (**b**) NO levels in different groups treated by LPS and ObEPS. (**c**–**f**) The impact of ObEPS on the transcription levels of IL-10 (**c**), TNF-α (**d**), IL-1β (**e**), and IL-6 (**f**) in RAW264.7 cells. Data are presented as mean ± SD (n = 4). Statistical significance: * *p* < 0.05; ** *p* < 0.01; *** *p* < 0.001; **** *p* < 0.0001.

To further investigate the biological activity and biocompatibility of ObEPS, we first assessed its potential cytotoxicity on the RAW264.7 macrophages. As shown in Figure 3a, ObEPS treatment at concentrations ranging from 25 to 200 μg/mL did not significantly affect cell viability compared to the untreated control group, indicating that ObEPS is non-toxic to macrophages at the tested doses.

Subsequently, we evaluated the anti-inflammatory potential of ObEPS by examining its effect on LPS-induced nitric oxide (NO) production in RAW264.7 cells. As depicted in Figure 3b, stimulation with LPS significantly increased NO levels, whereas ObEPS treatment dose-dependently suppressed NO production. Notably, the 200 μg/mL ObEPS group showed the most pronounced inhibition of NO, with a significant reduction compared to the LPS group, suggesting strong NO-scavenging and inflammation-attenuating effects of ObEPS.

To further explore the underlying anti-inflammatory mechanism, we measured the expression levels of pro-inflammatory and anti-inflammatory cytokines using qRT-PCR. As shown in Figure 3c–f, LPS stimulation markedly upregulated the mRNA expression of TNF-α, IL-1β, and IL-6, while downregulating the anti-inflammatory cytokine IL-10. Treatment with ObEPS at increasing concentrations reversed these changes in a dose-dependent manner. Specifically, ObEPS significantly upregulated IL-10 expression (Figure 3c) and suppressed TNF-α (Figure 3d), IL-1β (Figure 3e), and IL-6 (Figure 3f) mRNA levels. The LPS + 200 μg/mL ObEPS group exhibited the most robust modulatory effects, with statistically significant difference compared to the LPS group. These findings collectively demonstrate that ObEPS exerts potent anti-inflammatory effects without cytotoxicity, likely by modulating the expression of key inflammatory mediators in LPS-activated macrophages.

### 3.4. ObEPS Suppresses LPS-Induced Inflammation via Modulation of the NO Pathway

**Figure 4:**
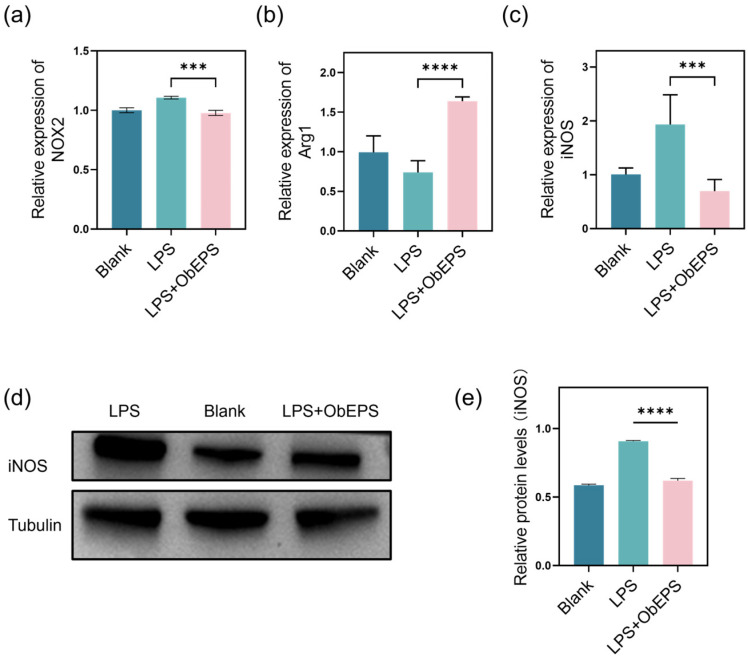
**ObEPS inhibits the expression of NOX2, Arg1, and iNOS in LPS-treated macrophages.** (**a**–**c**) Relative mRNA expression levels of NOX2, Arg1, and iNOS in the treated macrophages. The expression levels were evaluated by qPCR. (**d**) Representative Western blot images showing iNOS protein levels. Tubulin was used as a loading control. (**e**) Quantification of the iNOS protein levels relative to tubulin. Data are presented as mean ± SD (n = 4). Statistical significance: *** *p* < 0.001; **** *p* < 0.0001.

Given that ObEPS significantly inhibited NO production and reduced the mRNA expression of key pro-inflammatory cytokines in LPS-stimulated RAW264.7 macrophages (Figure 3), we further investigated whether this anti-inflammatory effect was mediated through the nitric oxide (NO) signaling pathway. Specifically, we examined the expression of genes closely related to NO synthesis and macrophage polarization, including NOX2, Arg1, and iNOS. As shown in Figure 4a, LPS stimulation slightly increased NOX2 expression, a marker of oxidative stress and M1 macrophage activation, whereas LPS + ObEPS treatment significantly suppressed this upregulation, suggesting a potential role in attenuating oxidative inflammatory responses. In contrast, the expression of Arg1, a classical marker of M2-type anti-inflammatory macrophages, was significantly elevated following LPS + ObEPS treatment, indicating that ObEPS may promote M2 polarization and a resolution-phase immune phenotype (Figure 4b). Moreover, the mRNA level of iNOS, a key enzyme responsible for excessive NO production during inflammation, was markedly increased in the LPS group but significantly reduced after ObEPS addition (Figure 4c). To validate this finding at the protein level, we conducted Western blot analysis of iNOS. The results demonstrate that ObEPS significantly suppressed the LPS-induced iNOS protein expression (Figure 4d), consistent with the qPCR results. Densitometric quantification confirmed a statistically significant reduction in iNOS protein levels (Figure 4e). Collectively, these results suggest that ObEPS exerts its anti-inflammatory effects at least in part through modulation of the NO pathway, including the suppression of NOX2 and iNOS, together with the upregulation of Arg1. This regulatory effect on macrophage phenotype and nitric oxide metabolism provides mechanistic insight into the immunomodulatory properties of ObEPS.

### 3.5. ObEPS Enhances Antifungal Efficacy and Reduces Renal Inflammation During Systemic C. albicans Infection

**Figure 5:**
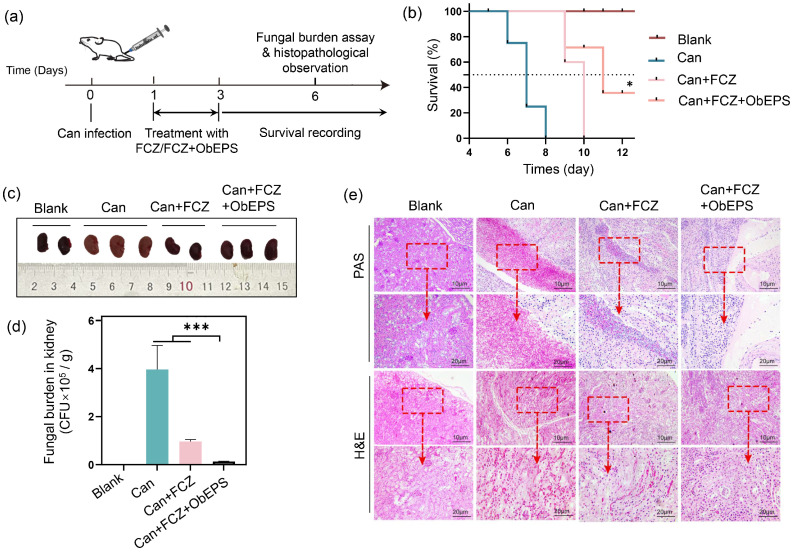
**Inhibition of mouse death and kidney inflammation by ObEPS after *C. albicans* systemic infection.** (**a**) Experimental timeline of systemic *C. albicans* infection and treatment. (**b**) Survival curves of mice in each group over 12 days. (**c**) Representative kidney images showing gross pathology on day 6. (**d**) Fungal burden in kidneys quantified by CFU counts. (**e**) Histological analysis of kidney sections stained with PAS and H&E. Data are presented as mean ± SD (n = 10). Statistical significance: * *p* < 0.05; *** *p* < 0.0001.

To further evaluate the in vivo anti-inflammatory and therapeutic potential of ObEPS, we established a murine model of systemic candidiasis by intravenous injection of *C. albicans*, followed by treatment with fluconazole alone or in combination with ObEPS (Figure 5a). Mice were monitored for survival, fungal burden, and kidney pathology. As shown in the survival curves (Figure 5b), untreated mice infected with *C. albicans* (Can) exhibited rapid mortality, with all animals succumbing by day 8. Treatment with FCZ moderately prolonged the survival time, but the mice failed to survive more than 10 days. In contrast, the combination of FCZ and ObEPS (Can + FCZ + ObEPS) significantly enhanced survival outcomes compared to FCZ alone, with 40% mice surviving after 12 days. This indicates a potential synergistic effect between ObEPS and antifungal therapy on the treatment of fungal systemic infection.

The kidney fungal burden and inflammation were then evaluated. Quantification of the fungal burden in kidney tissue (Figure 5d) demonstrated a high fungal load in the Can group (3.8 × 10^5^ CFU/g). The fungal burden was significantly reduced to 0.9 × 10^5^ CFU/g by the FCZ treatment, and further to 0.1 × 10^5^ CFU/g kidney by ObEPS + FCZ. In the in vitro antifungal assay, ObEPS alone had no obvious impact on the fungal growth (Figure S2). Both the Can + FCZ + ObEPS group and the Can + FCZ group showed significant differences compared with the Can group. However, there was no significant difference between the Can + FCZ + ObEPS and Can + FCZ groups (Figure S2), suggesting that ObEPS alone does not exert direct antifungal activity in vitro. Nevertheless, in vivo, its anti-inflammatory properties may help to reduce infection-associated tissue damage, thereby indirectly enhancing the therapeutic efficacy of fluconazole.

**Figure 6:**
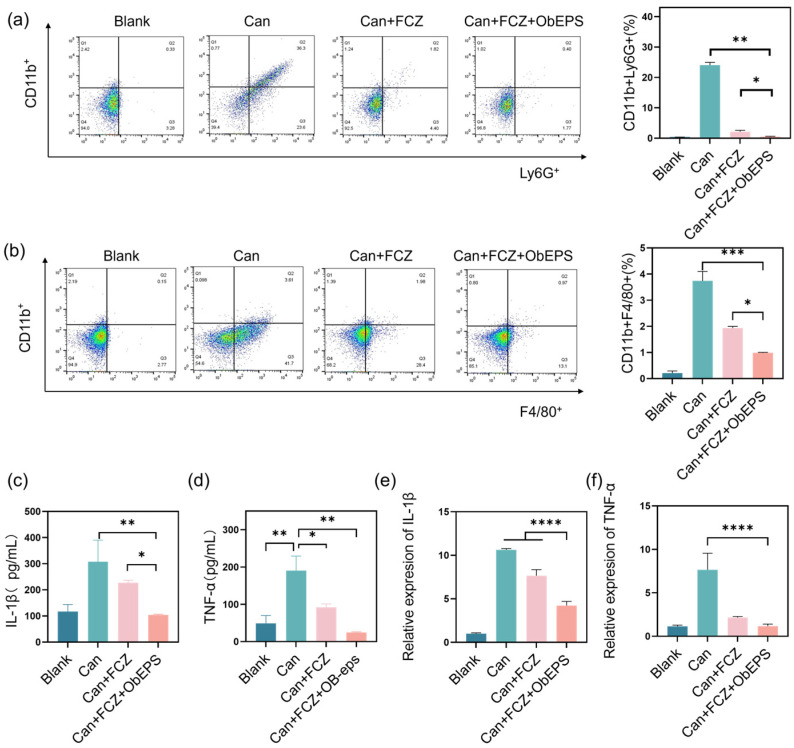
**Attenuation of immune cell accumulation and pro-inflammatory cytokine production by ObEPS in the infected mice.** (**a**) The quantification of CD11b^+^Ly6G^+^ neutrophils in kidney tissues (**b**) The quantification of CD11b^+^F4/80^+^ macrophages in kidney tissues. (**c**) ELISA analysis of IL-1β levels in serum. (**d**) ELISA analysis of TNF-α levels in serum. (**e**) Relative IL-1β mRNA expression in kidney tissue. (**f**) Relative TNF-α mRNA expression in kidney tissue. The asterisks indicate a significant difference between the two groups. Statistical significance: * *p* < 0.05; ** *p* < 0.01; *** *p* < 0.001; **** *p* < 0.0001.

Moreover, gross morphological examination of kidneys revealed that the Can group exhibited visibly enlarged and white kidneys, suggestive of severe fungal invasion and inflammation. In contrast, mice treated with FCZ or FCZ + ObEPS displayed kidneys with more normal morphology (Figure 6c). Histopathological analysis of kidney tissue further showed that the mice receiving the FCZ + ObEPS treatment maintained the intact tissue morphology, with a distributed population of immune cells and less fungal colonization (Figure 6e). The Can group showed extensive fungal infiltration, necrotic lesions, and inflammatory cell infiltration. In contrast, the Can + FCZ + ObEPS group exhibited markedly reduced fungal presence and tissue damage, with more intact renal architecture and reduced inflammation compared to the Can + FCZ group alone. These results indicate that ObEPS may enhance the antifungal drug efficacy. Together, these results indicate that ObEPS significantly enhances the therapeutic efficacy of FCZ in systemic candidiasis, potentially by synergizing fungal clearance and alleviating inflammation-associated renal damage.

### 3.6. ObEPS Suppresses Pro-Inflammatory Macrophage Polarization In Vivo

Given that ObEPS is a high-molecular-weight heteropolysaccharide composed mainly of Gal, Glc, Man, and Glc-UA (Figure 2a), we hypothesized that its unique branched structure and acidic sugar content may interact with pattern recognition receptors to modulate innate immune responses. To explore this, we investigated whether ObEPS could influence macrophage polarization during systemic candidiasis. Flow cytometry analysis revealed a substantial accumulation of pro-inflammatory Ly6G^+^ neutrophils (CD11b^+^Ly6G^+^) and inflammatory macrophages (CD11b^+^F4/80^+^) in the kidneys of *C. albicans*-infected mice (Can) (Figure 6a,b). FCZ treatment partially reduced this infiltration, but the combination of FCZ and ObEPS significantly suppressed both neutrophil and macrophage recruitment. This suggests that ObEPS exerts an immunomodulatory effect beyond its antifungal activity.

To further confirm its role in dampening the inflammatory environment, we quantified IL-1β and TNF-α secretion in kidney homogenates (Figure 6c,d) and serum (Figure 6e,f), both of which were markedly reduced in the Can + FCZ + ObEPS group compared to controls. Taken together, these findings demonstrate that ObEPS not only facilitates fungal clearance and reduces tissue damage, but also reprograms the inflammatory immune landscape by attenuating the polarization of macrophages toward a pro-inflammatory phenotype. The immunosuppressive capacity of ObEPS may be attributed, at least in part, to its acidic sugar residues and conformational properties, which potentially interact with macrophage surface receptors to trigger a regulatory signaling cascade.

## 4. Discussion

The opportunistic fungal pathogen *C. albicans* remains a major cause of invasive candidiasis, characterized by severe tissue inflammation and life-threatening organ dysfunction [29,30]. The kidneys are particularly vulnerable sites for fungal colonization and hyphal invasion, which exacerbates local immune responses and tissue injury [31]. Hyphal invasion disrupts epithelial integrity and triggers the release of damage-associated molecular patterns (DAMPs), which activate pattern recognition receptors, such as Toll-like receptors (TLRs) and C-type lectin receptors (CLRs) on resident and infiltrating immune cells. This engagement drives robust production of pro-inflammatory cytokines (e.g., TNF-α, IL-1β, IL-6) and chemokines, leading to massive neutrophil recruitment and reactive oxygen species (ROS) generation [32,33,34]. While the innate immune system plays a critical role in containing fungal proliferation, excessive or dysregulated inflammation often results in collateral damage, underscoring the need for therapeutic strategies that balance antifungal activity with immunomodulation.

Furthermore, polysaccharides have been shown to alleviate inflammation by inhibiting the production and release of key pro-inflammatory cytokines, including TNF-α, IL-1β, and IL-6 [35,36,37]. They can also downregulate the expression of inflammatory mediators, such as iNOS and COX-2 [38,39], thereby reducing excessive inflammatory responses and protecting tissues from further damage [40]. Previous studies have shown that various fungal and plant-derived polysaccharides can effectively suppress iNOS expression and NO production by modulating key inflammatory pathways in macrophages, such as through ceramide–PP2A signaling or the inhibition of the NF-κB and MAPK pathways [41,42]. In addition to these mechanisms, some polysaccharides have been reported to influence the STAT and Nrf2/HO-1 pathways, contributing to a broader anti-inflammatory profile and enhanced cellular antioxidant defense [43,44]. For example, β-glucans from *Inonotus obliquus* promoted M1 polarization and iNOS/TNF-α [45,46]. In this study, our findings revealed that ObEPS attenuated LPS-induced inflammatory signaling in vitro, as evidenced by reduced expression of the key inflammatory genes and proteins. Given that LPS stimulation is a well-established model for triggering innate immune activation, the observed effect of ObEPS suggests that it can interfere with upstream signaling events, potentially through modulation of TLR4-dependent pathways, suppression of IκBα degradation, or attenuation of MAPK phosphorylation [47,48]. In addition, polysaccharides containing amino sugars (GlcN and GalN) are known to exert protective roles by modulating immune responses and alleviating inflammation, partly through the regulation of cytokine production and antioxidant pathways [49,50,51]. Collectively, these chemical features also contribute to the distinctive biological activities of polysaccharides, including antitumor, antioxidant, immunomodulatory, anti-inflammatory, hepatoprotective, cardioprotective, and anti-aging properties.

This implies that ObEPS may act through multiple pathways to dampen excessive immune activation, thereby minimizing tissue damage associated with fungal invasion. Unlike conventional antifungal agents that directly inhibit or kill the pathogen, ObEPS provides a dual benefit by enhancing host defense mechanisms and curbing harmful inflammatory cascades. In a nutshell, our work highlights the potential of polysaccharide-based therapies as complementary approaches to conventional antifungal regimens. Within the scope of our experimental model, these polysaccharides demonstrated the immunomodulatory effect, which may contribute to the improved control of invasive candidiasis. However, the present study was limited to the in vitro and animal experiments. Its exact mechanisms, optimal dosages, and safety profiles in humans remain to be determined. Nevertheless, our study offers a strategy to manage invasive candidiasis and to reduce the risk of inflammation-driven organ injury.

## 5. Conclusions

In conclusion, this study developed a novel polysaccharide-based therapeutic strategy for treating systemic *C. albicans* infections. In a systemic *C. albicans* infection model, treatment with ObEPS alleviated kidney inflammation and prolonged the survival of infected mice. This work highlights the potential of the EPS from *O. brasiliensis* as a promising candidate for alleviating inflammation associated with both bacterial LPS and systemic *C. albicans* infection through its significant immunomodulatory activity. Although ObEPS exhibited obvious anti-inflammatory effects, its efficiency in the treatment of systemic infections remains to be improved. Future studies will focus on combining this EPS with nanomaterials or other delivery systems to further develop novel anti-inflammatory and anti-infection therapeutic strategies.

## Data Availability

The original contributions presented in this study are included in the article/supplementary material. Further inquiries can be directed to the corresponding author.

## Supplementary Materials

The following supporting information can be downloaded at https://www.mdpi.com/article/10.3390/microorganisms13092037/s1, Figure S1: ^1^H-Nuclear Magnetic Resonance (HNMR) spectrum of the purified polysaccharide on 600 MHz NMR spectrometer; Figure S2: In vitro growth curves of *Candida albicans* over 12 h under different treatments; Table S1. Nucleotide primer sequences used for PCR amplification; Table S2: qPCR cycling program; Table S3: Monosaccharide composition analysis of ObEPS. The contents of each monosaccharide are expressed as micrograms per milligram of sample.
