# Supplementary material for: Anti-Inflammatory Potential of Extracellular Polysaccharide from the Moss Endophyte Ovatospora brasiliensis During Pathogen Infection
Source: Microorganisms. 2025 Aug 31;13(9):2037. doi: 10.3390/microorganisms13092037 (PMC12472852; doi:10.3390/microorganisms13092037)
Supplement: Supplementary file 1 [file microorganisms-13-02037-s001.zip › microorganisms-3751570-supplementary.pdf]

Supplementary Materials

Anti-inflammatory potential of extracellular polysaccharide from the moss endophyte *Ovatospora brasiliensis* in pathogen infection

Jiayue Yang [1], Ying Sun [1], Mingchun Li [1] and Qilin Yu [1,2,*]

National Key Laboratory of Intelligent Tracking and Forecasting for Infectious Diseases, College of Life Sciences, Nankai University, Tianjin 300071, China

E-mail: yuqilin@mail.nankai.edu.cn

Table S1. Nucleotide primer sequences used for PCR amplification

Primer	Sequence	
	Sense	Anti-Sense
β-actinF	TGTCCACCTTCCAGCAGATGT	AGCTCAGTAACAGTCCGCCTAGA
TNF-αF	TGCCTATGTCTCAGCCTCTTC	GAGGCCATTTGGGAACTTCT
IL-1β	TGAAGCAGCTATGGCAACTG	AGGTCAAAGGTTTGGAAGGA
IL-6	AAGTGCATCATCGTTGTTCATACA	GAGGATACCACTCCCAACAGACC
IL-10	TGTTCACAGGTGACTGCTCC	AGCCCATAGTGGAGTGGGAT

Table S2. qPCR cycling program in this study

Step	Temperature	Time	Cycles
Initial Denaturation	94°C	30 sec	1
Denaturation	94°C	5 sec	
Annealing	55°C	15 sec	40
Extension	72°C	10 sec	
Dissociation Stage	-	-	1

Table S3. Monosaccharide composition analysis of ObEPS. The contents of each monosaccharide are expressed as micrograms per milligram of sample (μg/mg). The total monosaccharide content was 350.57 μg/mg.

Sample Name	GalN μg/mg	GlcN μg/mg	Gal μg/mg	Glc μg/mg	Man μg/mg	Glc-UA μg/mg	Total weight μg/mg
ObEPS	38.1870	32.2247	119.5888	19.012	93.8389	47.7173	350.5687

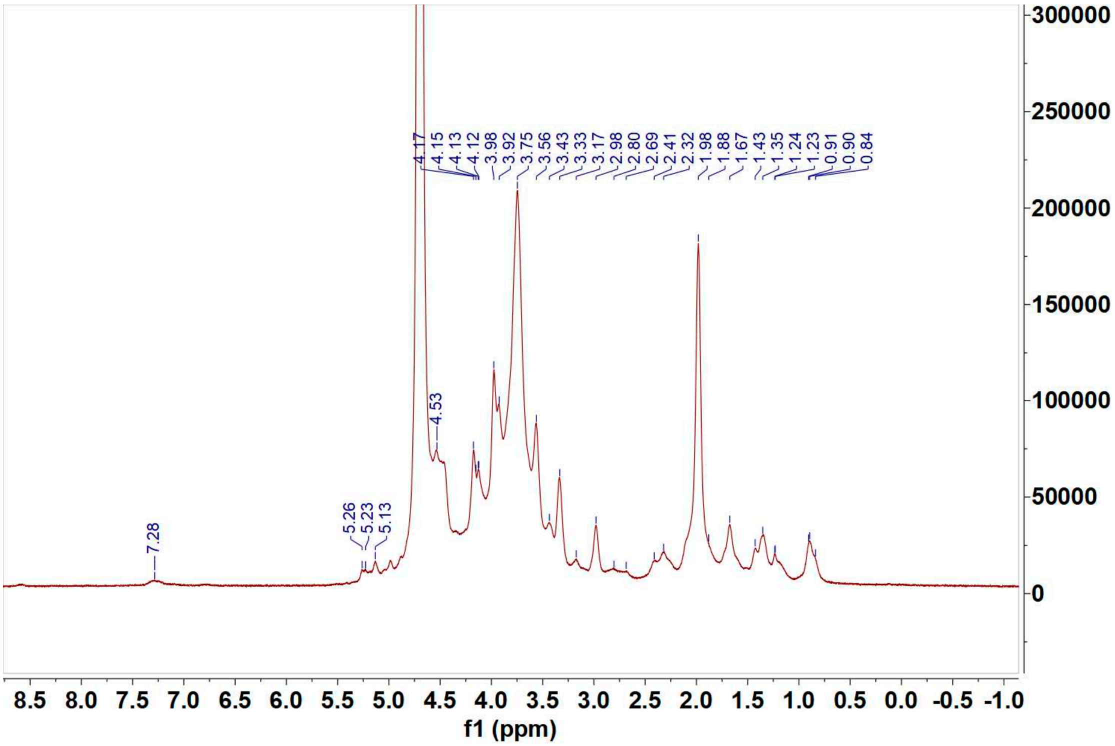

Figure S1. ^{1}H-Nuclear Magnetic Resonance (HNMR) spectrum of the purified polysaccharide on 600 MHz NMR spectrometer.

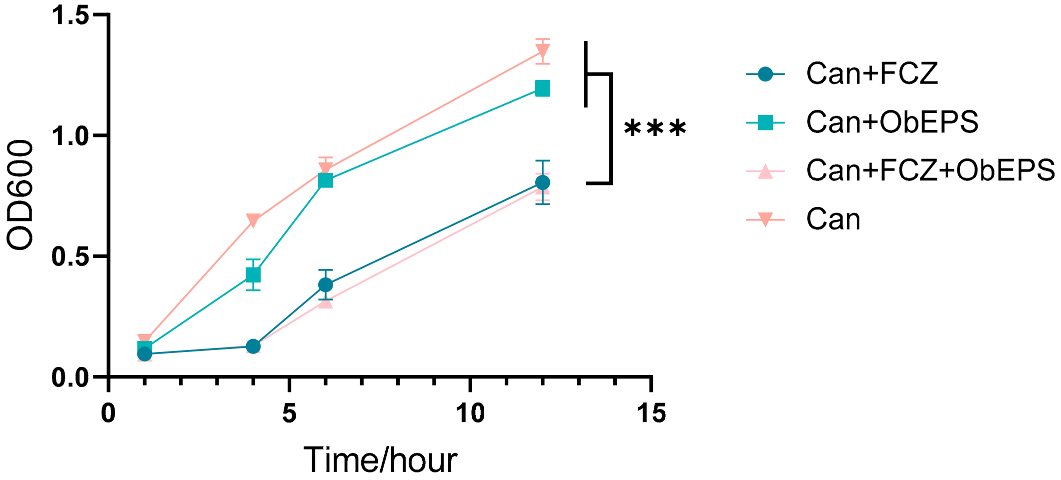

Figure S2. In vitro growth curves of *Candida albicans* over 12 h under different treatments. OD_{600} values were measured at 0, 4, 8, and 12 h for cultures treated with FCZ, ObEPS, their combination (FCZ + ObEPS), or untreated control (Can). Data are expressed as mean ± SEM (n = 4).
